# Efficacy of a Brief Intervention to Improve the Levels of Nutrition and Physical Exercise Knowledge Among Primary School Learners in Tshwane, South Africa: A Quasi-Experimental Study

**DOI:** 10.3390/ijerph21121592

**Published:** 2024-11-29

**Authors:** Morentho Cornelia Phetla, Linda Skaal

**Affiliations:** 1 Department of Human Nutrition & Dietetics, Sefako Makgatho Health Sciences University, Pretoria 0001, South Africa; 2 Department of Public Health, Sefako Makgatho Health Sciences University, Pretoria 0001, South Africa; linda.skaal@smu.ac.za

**Keywords:** intervention, learners, knowledge, physical activity, nutrition

## Abstract

The prevalence of childhood obesity in South Africa necessitates the design and implementation of interventions to improve the levels of physical activity and nutrition among school-age children. This study aimed to evaluate the efficacy of a brief intervention to improve knowledge regarding exercise and nutrition among primary school learners in Tshwane, South Africa, using a quasi-experimental single-group, pre–post-test design. A total of 86 primary school learners from two primary schools participated in the intervention, which was guided by the Analysis Grid for Obesity-Linked Environments (ANGELO). Descriptive statistics were analysed using the frequency distribution while inferential statistics used the *t*-test to compare the means of nutrition and exercise knowledge pre- and post-intervention. All primary school learners were able to access the nutrition and exercise information. The mean nutrition knowledge (pre- and post-test) was 0.914 and the mean exercise knowledge (pre- and post-test) was 0.8464. The primary school learners’ levels of knowledge on diet and physical exercise were improved by the brief intervention. It is recommended that this initiative be supported, continued, and duplicated in schools throughout Tshwane.

## 1. Introduction

According to the World Health Organization (WHO), childhood obesity remains a concerning public health challenge. It is reported that over 390 million children and adolescents aged 5–19 years are overweight and 160 million children are obese globally [1]. Studies show that childhood obesity impacts quality of life (QoL), as it leads to high rates of early morbidity and mortality [2]. Children who are obese are likely to present with poor physical well-being, psychological problems (e.g., low self-esteem), emotional problems, poor academic performance, medical consequences, comorbid conditions, and poor QoL overall [3,4,5,6].

Given the prevalence of childhood obesity, there is a need to implement and evaluate brief interventions to improve the levels of physical activity and nutrition among primary school learners. Brief interventions, which are techniques used to initiate a change in unhealthy behaviours, have been used over the years and found to be effective in some instances [7,8].

School health programmes (SHPs) are necessary to promote the health and well-being of primary school learners. SHPs collaborate with health promotion, education, and communication within and between sectors. In 2013, the WHO proposed the implementation of health-promoting schools (HPSs) worldwide. When implemented correctly, the SHP has positively impacted learners in developed countries such as Canada [9]. In Ghana, a healthy food environment policy was successfully implemented [10]. However, in developing countries, such as Malaysia and Iran, the implementation of SHPs has been reported to be unsuccessful [11,12].

Rossettie et al. (2018) [13] surveyed the impact of the national implementation of fruit and vegetable provisions and sugar-sweetened beverage (SSB) restrictions in the United States. The survey showed that the implementation of national school policies on fruit and vegetable consumption improved consumption, while the restriction of SSBs reduced consumption, thus improving dietary habits. A recent assessment of the health and economic effects of U.S. school meal standards shows that compliance with the implementation of diet guideline standards can improve dietary, childhood health, and chronic diseases [14]. A systematic and meta-analysis review conducted in the United States shows that dietitians and exercise practitioners play a key role in promoting positive lifestyle behaviors [15].

Programmes initiated in South Africa include the National School Nutrition Programme (NSNP) [16], which has not yielded significant changes in nutrition and physical activity (PA) among learners. The NSNP aims to provide nutritious meals to learners and teach them about healthy eating, but food suppliers are noncompliant, food is not prepared properly, and the menu does not include fruits and vegetables, as recommended by the South African Food-Based Dietary Guidelines (SAFBDGs).

The National Department of Health’s Strategy for the Prevention and Management of Obesity in South Africa 2023–2028 [17] emphasises a multisectoral, multidisciplinary approach and the creation of enabling environments for healthy eating and exercise, yet schools engage in unstructured obesity-prevention programs that are unsuccessfully implemented. Moreover, the action plans for the national strategy have yet to be implemented and monitored.

Other efforts to curb the rate of obesity in South Africa include guidelines and policies to help the Department of Basic Education (DBE), including the Tuck-Shop Operator Guidelines (2014) [18]. However, there is no proper monitoring and evaluation of the operations of tuck shops and street vendors around primary schools.

Despite the programmes mentioned above, a previous study by the researchers of the current study shows that learners in primary schools in Tshwane remain obese because of poor knowledge of physical activity (PA) and nutrition behaviours. In their initial study, learners reported that the time allocated for physical education (i.e., sports and exercise) is, at times, used for other academic purposes or for idling outside classrooms. Furthermore, teachers have reported difficulty in implementing exercises according to the Annual Teaching Plan (ATP) because they have not been trained on exercise prescription or only have access to inadequate sports equipment and facilities [19]. Based on these findings, the researchers designed and implemented a brief intervention to improve the levels of PA and nutrition knowledge among these learners.

This study developed and implemented a nutrition and exercise programme guided by the Analysis Grid for Environments Linked to Obesity (ANGELO) framework. This framework enabled the researchers to scan the behaviour, knowledge, and skill of learners related to PA and nutrition and then prioritise action plans according to the levels of importance and changeability. Through that, they developed a smart action plan to improve the levels of PA and nutritional knowledge. This method is unique in that it allowed the researchers to make immediate changes and restructure their intervention on the go. Studies show that an improvement in knowledge and the creation of an enabling environment yield significant changes in exercise and nutrition behaviour [20,21,22]. It is envisaged that regular and long-term implementation of exercise and nutrition programmes will reduce the prevalence of obesity and its consequences, such as lack of confidence, low self-esteem, poor cognitive development, poor psychology, and poor well-being.

## 2. Materials and Methods

A single-group, pre- and post-test quasi-experimental design was used in this study. This design uses the same individuals for both the control and intervention groups, meaning that knowledge was measured before and after the intervention on the same individuals, as illustrated below:

***O1(pre-test)***…………………***X1(intervention)***…………***02(post-test)*** (1)

In this study, two [2] primary schools based in Tshwane West District, South Africa, were enrolled in the intervention. A total of 86 primary school learners from grades 5 to 7 were enrolled in the study because of their level of understanding of concepts. The researchers included all learners in grades 5 to 7 whose parents had signed informed consent, and the learners assented to participate in the study. The baseline data were collected in 2021, and the post-test data were collected in 2022. The 86 learners who were included in the study were those who participated in the baseline study in 2021. In 2021 they included grades 4 to 7. In 2022, they only included grades 5 to 7, as grade 4 learners were not included during the baseline. The intervention was only implemented in two schools because of their proximity and the permission given by school principals.

Validity was guaranteed by consulting experts in nutrition and exercise to validate the contents of the educational materials. To avoid bias, the students had studied for a BSc (dietetics) or BSc (physiotherapy) within the last year were supervised by qualified professionals to carry out the activities. The intervention was implemented for three months by the researchers, a qualified dietitian, research assistants, and qualified physical instructors. The activities included compiling nutrition and exercise educational booklets and pamphlets, conducting exercise classes, distributing and hanging nutrition and exercise posters, observing learners’ eating habits, inspecting lunch boxes, and scanning school surroundings for obesogenicity.

### 2.1. Data Collection Instruments

**Figure 1:**
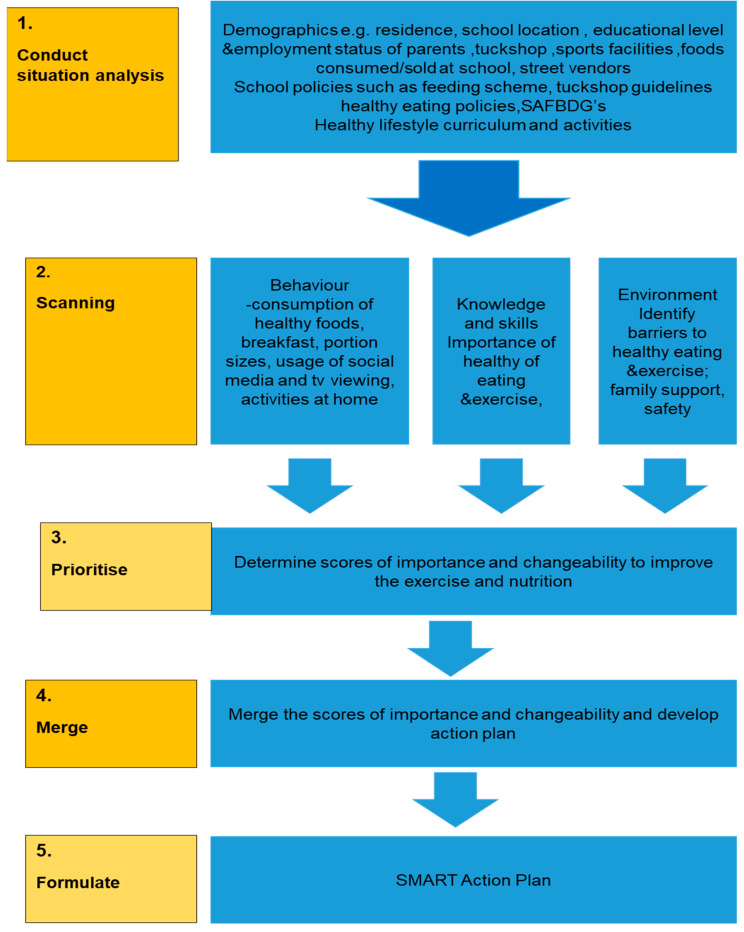
Operational diagram of the ANGELO framework adopted for this study.

The data collection instruments used in this research were pre- and post-researcher-administered questionnaires, anthropometrics, and an observation checklist. The data were collected during baseline and after three months. An observation checklist was used to scan school environments for obesogenic schools. The researchers used the ANGELO framework as a guide to develop an observation checklist that evaluated the school tuckshop, street vendors, food sold and consumed in the school and surrounding areas, food preparation methods, quantity of food eaten, physical activities, and advertisements around the school and sports facilities/play areas (Figure 1). The ANGELO framework was implemented as follows:

#### 2.1.1. Step 1: Conduct Situational Analysis

A situational analysis was conducted in the schools during the data collection period. The researcher and research assistants spent prolonged periods at the schools and its surroundings. A situational analysis consists of the physical, legislative, sociocultural, and economic domains. The physical domain consists of the buildings, amenities, facilities, built environment, and enclosed spaces. The legislative domain includes rules, legal guidance, statutory provisions, and political messages. The sociocultural domain consists of the attitudes, beliefs, perceptions, and norms in a community. The economic domain includes monetary costs, consequences, incentives, and taxes [23]. During the situational analysis, the following was observed:

*Physical domain*: This entailed analysis of the following: location, kilometres to Pretoria city centre, economic status of the people residing in the area, availability of sports facilities, availability of a tuckshop, type of foods sold, food items mostly consumed by the learners, and reasons for consumption of food sold by street vendors.

*Legislative domain*: This entailed the following: the availability of school policies; school compliance with policies; school feeding programme, how it operates, who benefits from it, the menu, time when meals are served, portion sizes, and food preparation methods. The availability of policies that govern the tuckshop and healthy eating around the school were reviewed, along with The Life Orientation and Life Skills curriculums. The availability and adherence to South African Food-Based Dietary Guidelines (SAFBGs) were also reviewed.

*Sociocultural domain*: This entailed observing and enquiring about the cultural foods eaten in the school and surrounding community.

*Economic domain*: This entailed questions about pocket money and how it was spent, as well as whether parents were working.

#### 2.1.2. Step 2: Scanning

Scanning includes observing behaviour, knowledge, and skills, as well as the environment. The following was scanned: consumption of healthy foods and lunchboxes during tea and lunch. Questions were asked about the frequency of social media use, the period of television viewing at home, and activities conducted after school.

Lunchboxes were observed to check whether parents were preparing and packing healthy foods and snacks. Learners were asked whether they knew about healthy eating and exercise.

Learners were asked whether their families encouraged them to partake in exercise. Questions were asked about safety, e.g., whether there was transport to take learners home after exercises or whether it was safe to walk alone.

#### 2.1.3. Step 3: Prioritise

Researchers focused on what was available in schools and what could be changed to improve nutrition and exercise. The researchers prioritised what was feasible by intervening in programmes that were already in existence.

#### 2.1.4. Step 4: Merge

The scores of importance and changeability were merged and the programme was developed.

#### 2.1.5. Step 5: Formulate

A SMART (specific, measurable, achievable, realistic/relevant, and time-bound) action plan was developed consisting of aims, objectives, and strategies. The plan was initiated to develop and implement a nutrition and exercise programme.

The researcher and trained research assistant administered a survey questionnaire to collect the data. The questionnaire was translated into the local language. Data were collected at baseline and again post-test using the same questionnaire (nutrition and exercise knowledge).

### 2.2. Data Analysis

The questionnaires were coded, and the information was entered into the Statistical Package for Social Sciences (SPSS) version 27 software for analysis. Descriptive statistics used frequency distribution, standard deviation, and means. Inferential statistics used a *t*-test to compare the mean knowledge pre- and post-test.

### 2.3. Brief Intervention

The programme was implemented for three months by the researcher, trained research assistants, and physical instructors (described in detail in supplementary Table S1). The intervention focused on healthy eating and exercise in the schools.

#### 2.3.1. Nutrition and Exercise Education for School Learners

Upon the initial assessment, learners’ health-related knowledge and practice were found to be poor. The intervention, therefore, focused on improving knowledge and practice by implementing the following activities during the Life Orientation and Life Skills periods within timeslots approved by the school management:

- Educational presentations on the benefits of healthy eating; the importance of consuming fruits, vegetables, and water; and the dangers of consuming unhealthy foods, such as sweetened, fatty, and junk foods. Nutrition education was mainly based on the SAFBDGs;
- For exercise: educational presentations focused on the benefits of exercise, exercise prescription, and injury prevention;
- To further enhance knowledge, researchers designed posters and booklets, as well as used food models.

#### 2.3.2. Nutrition and Exercise Materials

Before the implementation period, the researchers designed child-friendly posters and exercise and nutrition booklets. These posters and booklets were brightly coloured and had many pictures with brief, informative messages. The posters were hung and pasted on all grade 4 to 7 classroom walls and in the strategic areas of the schools where all learners could always see them.

The child-friendly information booklets were distributed to all learners so that they could learn more about nutrition and exercise. The teachers were also provided with soft copies of the materials for future use.

#### 2.3.3. Nutrition and Exercise Education for Teachers

The researchers selected Life Skills and Life Orientation teachers to improve their knowledge on the benefits of exercise and healthy eating. The reason for this selection was that they were the ones responsible for reinforcing life skills such as nutrition and exercise at schools. Teachers were trained on all aspects of the SAFBDGs guidelines and exercise knowledge using PowerPoint presentations, information booklets, and exercise demonstrations.

#### 2.3.4. Training of Voluntary Food Handlers (VFHs)

Voluntary Food Handlers were trained using training materials prepared by the researchers. The training emphasised the following:

- How to urgently substitute the menu in case of the delayed delivery of other food items on the menu;
- Food safety and hygiene;
- A demonstration on how to cook vegetables healthily;
- The use and implementation of the NSNP document.

#### 2.3.5. Launch of Sports Day Events

The researchers organised separate Sports Day events for the two schools in order to pilot the programme. The sports days were conducted for four days in each school over the three-month study period. On the sports days, the entire day was dedicated to sports activities and educational talks, and learners were informed to wear exercise clothes and to bring water bottles to school. The researchers purchased sports equipment such as hula hoops, skipping ropes, soccer balls, basketballs, and netballs. This equipment was used during the sports days and then donated to the schools.

The sports days were facilitated by dieticians and qualified physical activity instructors. The school learners were divided into smaller groups and assigned to different instructors who exposed learners to different types of exercises, verbally motivated them and explained the importance of exercising and sports nutrition. The trained research assistants screened learners for anthropometric measurements and calculated the BMI. The learners with lesser or greater BMI than normal were instructed to consult a dietitian in public or private health institutions for further intervention.

#### 2.3.6. Create Awareness About the NSNP Policy

We conducted a session with staff to create awareness regarding the following:

- SA tuckshop guidelines: copies of the DOE tuckshop guidelines were distributed;
- The importance of establishing a vegetable garden and healthy eating.

A summarised version of the NSNP policy was created in poster format and distributed to each participating school.

#### 2.3.7. Establishment of the Vegetable Garden

We facilitated the creation of a vegetable garden in collaboration with school management. We donated garden equipment, fertilisers, and vegetable seeds. The research assistants and general workers physically worked on the creation of a garden in each school.

## 3. Results

Table 1 indicates that the age range of the learners was between 9 and 16 years with a mean of 10.89 ± 7 years. The data indicate that half of the learners were girls and half were boys. Most (80.2%) of the learners stayed with their parents. More than half of the learners (65.1%) carried lunch boxes and pocket money (70.9%) to school. Most (75.6%) learners ate from the school feeding programme. More than half of the learners (54.7%) walked to school while some travelled by car (45.3%). Almost half of the learners (48.8%) had a normal body mass index (BMI). There was no significant difference between boys and girls regarding learners’ demographic characteristics; however, boys were thinner than girls.

Table 2 shows that all (100%) learners saw the posters about nutrition, and they all read what was written on the posters. They all indicated that they understood the information on the posters and that the content improved their knowledge of nutrition. All the learners reported that the booklets improved their nutrition knowledge. The results further indicate that all learners attended nutrition education classes. They all read and understood what was written in the nutrition booklet.

The table further indicates that all (100%) learners saw the posters about exercise and read what was written on them. All learners indicated that they understood the information on the posters and that the content improved their knowledge of exercise. All learners reported that the booklets improved their exercise knowledge. All learners attended exercise education classes and reported that they read and understood what was written in the booklets. All learners attended exercise education classes and understood the information presented. They all reported that exercise education improved their knowledge and that they were willing to exercise three times a week for 60 min. All learners participated in Sports Day and enjoyed the activities. They indicated that they benefited from the Sports Days and that they would implement what they had learned.

Table 3 shows the difference between the knowledge of the paired samples (nutrition and exercise) pre- and post-test. The paired samples test showed significant differences. The difference signifies that the nutrition and exercise programmes had a positive impact.

## 4. Discussion

The results show that an intense exercise and nutrition programme can help learners improve their knowledge, as shown in Table 3, where it was found that the mean scores differed positively between the pre- and post-tests. Previous studies have shown that exposure to educational materials, such as posters, pamphlets, and awareness campaigns, significantly improves knowledge [24,25]. Similarly, a study conducted in the UK showed that packaging education in activities such as Food a Fact of Life (FFL) kits was effective in improving knowledge and nutrition practices [26,27]. According to the results of the current study, it is clear that all learners were interested in the nutrition and exercise information they had. However, previous studies have shown that learners often ignore educational materials or claim that posters and pamphlets are too difficult to read and understand because of font sizes, extensive information, and the difficult language used [28,29]. To solve these shortcomings, our study developed user-friendly materials in their local language that are easy to understand, which explains why learners achieved high scores on the usability of all materials.

Our study used various approaches, including posters, pamphlets, and educational talks with pictures, to attract more interest and achieve deeper understanding on the part of learners. These different approaches have been reported to be effective training tools in countries such as Indonesia, where it has also been found that training schoolchildren in nutritional education through videos and posters had a positive impact on nutrition knowledge, attitudes, and actions [30]. These studies show the importance of using different materials, such as posters, brochures, and videos, to convey both nutritional and physical activity education messages to learners, bearing in mind that learners have unique reactions depending on the platform of communication. The West Tshwane District is characterised by a low to medium socio-economic status, with a low level of education; therefore, it was important to use and translate the educational materials into the local language to include learners who do not understand English, and physical education teachers conducted their training in the local language, which further enhanced the usability of our training materials. The training materials used in our study have been used in other studies such as those by Dobbins et al. [31], who found that printed materials such as posters and brochures positively increased physical activity.

Our study was carried out in an area with low socio-demographic conditions, characterised by the fact that no educational materials were found in the school environment [19], which meant that we had to transform the physical environment with posters and issue educational brochures to the learners to improve both nutritional and physical activity knowledge. Visual materials such as posters and colourful brochures helped children focus and retain their interest in the content. In developed countries, studies have shown that all knowledge materials are easily available in physical and digital formats [32,33], which means that educational resources should not be limited to posters and brochures but should be formatted and made available electronically. As South Africa has experienced a change in its digital systems because of the advent of the Fourth Industrial Revolution (4IR), innovative teaching methods, such as the use of electronics and simulations, should be implemented at the primary school level to advance the transmission of messages to learners.

This study revealed that all learners attended nutrition and physical activity education classes that were organised and delivered by the researchers and that they understood the information presented to them, especially because the researchers used the local language in the development and delivery of content, and that all messages were simplified with locally produced food posters. Initially, our evidence shows that learners consumed unhealthy foods [19], and, after the intervention, they understood the importance of eating healthy foods, i.e., their nutritional knowledge improved. All learners reported that nutrition education improved their knowledge, and all reported that they were willing to buy and eat healthy food every time they had enough money to buy it.

As reported in the current study, we found that more than two-thirds of learners carry pocket money to school, so equipping them with nutritional knowledge would increase the informed choice of what to buy. We will continue to work with schools to help improve the school environment to reduce their obesogenicity. Like other studies, there are benefits to nutritional interventions, as they report a significant improvement in nutrition knowledge after the implementation of nutrition education [34,35,36,37]. With all the reports, it would also be beneficial for DBE to collaborate with the National Nutrition Directorate to develop and deploy similar resources over the long term. From our previous study [19], we found that the majority of learners did not participate in physical activity on school grounds even though both schools had sports grounds. This led researchers to devise ways to re-introduce sports in schools. Although sports activities were stopped during 2020 due to the COVID-19 pandemic lockdown restrictions, these restrictions were already relaxed in 2021–2022 during data collection; however, it was worrying that both schools were still not active in reintroducing learners to sporting activities because there were no structured physical activities in both schools after the COVID-19 pandemic. Therefore, we introduced more exciting activities such as hula-hooping, skipping rope, and various ball activities, such as volleyball, netball, and football, to the two schools. To generate more interest, we have teamed up with qualified training instructors to educate and demonstrate safer ways of exercising among learners, further increasing participation in physical activity.

The results of this study show that the levels of knowledge and interest in physical activity has improved, as a result, learners report that they have begun exercising regularly after exposure. Similarly, studies conducted among school children in Italy yielded the following benefits: students became passionate about physical activity, improved concentration in class, showed improvements in attitudes and interest in sports, and reduced anxiety [38]. Other studies have also shown that exposure to physical activity interventions improves participation and physical fitness among school children [31,39,40,41]. All these studies further demonstrate that the benefits of exposing learners to exercise interventions at the beginning of their lives extend throughout their lives. It is of interest that learners in these two primary schools began to exercise regularly, and the donated exercise equipment has contributed to increasing the level of participation and introduced an additional variety of accessible physical activities to the schools, as mentioned above.

The Global Strategy for Diet, Physical Activity, and Health (2018) recommends that learners participate in physical activity several days a week [42], which was also considered and highlighted by the physical instructors of the current study. In terms of sustainability, we emphasised the importance of using cost-effective measures and materials for learners such as running and cycling as part of their physical education. We encouraged learners to participate in physical activities outside school and continue to exercise at home or around their neighbourhoods with family members to maximise the results of healthy food and exercise

### 4.1. Study Limitations

The brief intervention was implemented only in two schools in Tshwane West, Gauteng, due to a limited time frame. The researchers had limited time to complete the studies. The intervention was conducted on a few learners in two schools due to logistic reasons and ease of control of the intervention. Furthermore, since the intervention was conducted for a short period, researchers were unable to ask learners questions about any changes in their practices.

### 4.2. Practical Implications

The programme was piloted in two schools in Tshwane West due to limited resources and to establish its effectiveness. The programme proved to be well-received by learners and school staff, as it is easy to follow and sustain. It is recommended that the same programme be rolled out to all primary schools in Tshwane. Independent research can be conducted to validate this programme.

The DBE should formally train Life Skills and Life Orientation teachers from each school so that they will be able to sustain the programme and should consider hiring full-time nutrition experts, either dietitians or nutritionists, for each school. Nutrition experts will be responsible for nutrition-related matters including the NSNP. It is further recommended that schools embark on a drive to educate children about the benefits of consuming balanced meals. The DBE should consider hiring a part-time physical instructor for all matters relating to exercises and sporting codes at each school. The parents should be sensitised to the programme so that they can support the learners at home. Impact studies will be conducted by postgraduate students in 2025 from our university.

## 5. Conclusions

A brief intervention was developed and implemented in the Tshwane West District for three months. The intervention was well-received by the learners and school staff, as it was easy to follow and maintain. The intervention was effective in improving physical activity and nutrition knowledge among the learners. It is recommended that the same programme be implemented in all primary schools in Tshwane. Independent research can be conducted to validate this programme.

## Figures and Tables

**Table 1:** Learners’ demographic characteristics.

Variable		Boys = (n%)	Girls = (n%)	*p*-Value
**Age groups**	<11 years	27 (63)	32 (74)	0.245
	>11 years	16 (37)	11 (26)	
**Learners stay**	Parents	36 (84)	33 (77)	0.430
	Others	7 (16)	10 (23)	
**Carries lunchbox**	Yes	35 (81)	32 (74)	0.143
	No	8 (19)	11 (26)	
**Eats via a feeding scheme**	Yes	32 (74)	33 (77)	0.881
	No	11 (26)	10 (23)	
**Carries pocket money**	Yes	33 (77)	28 (65)	0.650
	No	10 (33)	15 (35)	
**Mode of transport**	Car	21 (49)	18 (42)	0.650
	Walk	22 (51)	25 (58)	
**BMI**	Obese	1 (2)	3 (7)	0.151
	Overweight	3 (7)	1 (2)	
	Normal	17 (40)	25 (58)	
	Thinness	22 (51)	14 (33)	

**Table 2:** Learners’ ability to access educational materials for nutrition and exercise information.

Statement Related to Posters—Nutrition Booklet and Nutrition Education Used in the Implementation of Nutrition Programme	Yes
Posters (n = 86)	
Did you see posters about nutrition and exercise?	100%
Did you read what is written on the posters?	100%
Did you understand the information on the posters?	100%
Did the posters improve your knowledge of nutrition and exercise?	100%
**Nutrition booklet (n = 86)**	
Did you receive the nutrition and exercise information booklet?	100%
Did you read what is written in the nutrition and exercise booklet?	100%
Did you understand the information in the nutrition and exercise information booklet?	100%
Did the information in the booklet improve your nutrition and exercise knowledge?	100%
**Nutrition education (n = 86)**	
Did you attend nutrition and exercise education classes?	100%
Did you understand the information presented during nutrition and exercise education?	100%
Did nutrition and exercise education improve your knowledge?	100%
Did you start eating healthy foods daily?	100%
Are you willing to exercise 3 times a week for 60 min?	100%
**Sports Day (n = 86)**	
Did you enjoy the activities of the sports day?	100%
Did you benefit from sports day?	100%
I have started to exercise regularly.	100%

**Table 3:** Paired samples test (paired differences).

	Mean	Std Deviation	Std. Error Mean	95% Confidence Interval of the Difference				Significance	
				Lower	Upper	t	df	One-Sided p	Two-Sided p
Pair 1 Nutrition knowledge (pretest) Nutrition knowledge (post-test)	−5.34884	3.31047	0.35698	−6.05860	−4.63907	−14,984	85	<0.001	<0.001
Pair 2 Exercise knowledge (pretest) Exercise knowledge (post-test)	−2.52326	1.69238	0.18249	−2.88610	−2.16041	−13,826	85	<0.001	<0.001

## Data Availability

The data presented in this study are available upon request from the corresponding author.

## Supplementary Materials

The following supporting information can be downloaded at: https://www.mdpi.com/article/10.3390/ijerph21121592/s1, Table S1: Action Plan for the nutrition and exercise programme.
