# Supplementary material for: Efficacy of a Brief Intervention to Improve the Levels of Nutrition and Physical Exercise Knowledge Among Primary School Learners in Tshwane, South Africa: A Quasi-Experimental Study
Source: Int J Environ Res Public Health. 2024 Nov 29;21(12):1592. doi: 10.3390/ijerph21121592 (PMC11675107; doi:10.3390/ijerph21121592)
Supplement: Supplementary file 1 [file ijerph-21-01592-s001.zip › ijerph-3247464-supplementary.pdf]

Table S1. Action Plan for the nutrition and exercise programme

**Goal**: To prevent and reduce obesity amongst primary school learners in Tshwane West District, Gauteng, South Africa			
**Objective 1**: To increase the consumption of fruits and vegetables amongst all Grade 4 to 7 primary school learners in two identified primary schools through nutrition education and poster development by the end of 2022.			
**Activity**	**Time frame /Frequency**	**Resources**	**Responsible person**
Educate all Grade 4 to 7 children about the importance of fruits and vegetables	Once a week per class for 6 months	Dietitian Research assistants	Researcher
Develop nutrition posters that display the importance of consuming different fruits and vegetables	1 month		
Distribute posters and nutrition pamphlets in all Grade 4 to 7 classrooms	1 month	Funding – development of posters<br>Transport to schools	
Establish the vegetable garden, vegetables will be used for school feeding	1 month	Funding for garden seeds &	Researcher

		gardening equipment<br><br>Manpower	
**Objective 2:** To reduce consumption of SSB ,sugar , sugary foods and junk foods amongst all Grade 4 to 7 primary school learners two identified primary schools through nutrition education and poster development by end of 2022			
**Activity**	**Time frame /Frequency**	**Resources**	**Responsible person**
Educate all Grade 4 to 7 children about the dangers of SSB ,sugar , sugary foods and junk food consumption	Once a week per class for 6 months	Dietitian Research assistants<br>Funding – development of posters<br><br>Transport to schools	Researcher

Develop nutrition posters that display the dangers of SSB, sugar, sugary foods, and junk foods	1 month		
Distribute posters and nutrition pamphlets in all grade 4 Grade 4 to 7 classrooms	1 month		
**Objective 3:** To To increase the consumption of water amongst all Grade 4 to 7 primary school learners in two identified primary schools through nutrition education and poster development by end of 2022			
**Activity**	**Time frame /frequency**	**Resources**	**Responsible person**
Educate all Grade 4 to 7 children about the importance of water consumption	Once a week per class for 6 months 1 month	Dietitian<br>Research assistants<br>Funding – development of posters<br>Transport to schools	Researcher
Encourage all Grade 4 to 7 children to bring water bottles to school every day			
Develop and distribute posters that display the importance of water and the frequency of drinking water			
**Objective 4:** To improve nutrition and exercise knowledge amongst all Grade 4 to 7 primary school learners in two identified primary schools using different educational methods by the end of 2022			

Activity	Time frame /frequency	Resources	Responsible person
Apply different methods of educating learners about nutrition e.g. quiz, lessons, posters, role play	Once a week per class for 6 months	Dietitian Research assistants<br><br>Food models Life Orientation and Life Skills teachers<br><br>Funding – development of teaching materials<br><br>Transport to schools<br><br>Exercise instructors	Researcher
Apply different methods of educating learners about exercise e.g. lessons, quizzes, demonstrations			
Development of nutrition materials such as posters, pamphlets			

Distribution of posters in all the classrooms and key strategic areas	1 month	Research assistants<br>Life Orientation<br>and Life Skills teachers	
Educate and demonstrate a healthy lunch box	1 month	Dietitian Research assistants	
**Objective 5**:To improve exercises amongst all Grade 4 to 7 primary school learners in the two identified primary schools through design and development of structured physical education activities by the end of 2022			
**Activity**	**Time frame /frequency**	**Resources**	**Responsible person**
Review and discuss the physical education activities in the DBE Annual Teaching Program (ATP) with Life Orientation and Life Skills teachers	1 month	Life Orientation and Life Skills teachers<br><br>Physical instructor	Researcher
Review and discuss the physical education activities in the DBE Annual Teaching Program (ATP) with the exercise instructor	1 month		
Develop and design structured physical education activities guided by ATP	1 month		

Implement the physical education activities during Life Orientation and Life Skills classes	Once a week per class for 6months	Research assistants	
Donate cost-effective exercise equipment's for the school	1 month	Funding	
**Objective 6**: To improve the school feeding program and food preparations by providing in-service training to all voluntary food handlers (VFH) in the two identified schools by the end of 2022.			
**Activity**	**Time frame /frequency**	**Resources**	**Responsible person**
Train VFH how to prepare food in a healthy way	Twice a week for the period of 6 months	Dietitian NSNP document Dietitian Research assistants	Researcher
Train and demonstrate to VFHs how to prepare healthy vegetables			
Educate the VFHs on how to plan a substitute menu			
Provide in-service training to voluntary food handlers about NSNP			
**Objective 7**: To improve nutrition and exercise knowledge amongst Grade 4 to 7 teachers in the two identified schools by conducting in-service training sessions by end of 2022			
**Activity**	**Time frame /frequency**	**Resources**	**Responsible person**

Review and discuss nutrition topics as stipulated in the Life Orientation and Life Skills curriculum	Once a week for 6months<br><br>March 2022<br><br>March 2022	Exercise instructor<br><br>Research assistants<br><br>Life Orientation and Life skills teachers<br><br>Funding – Printing of teaching materials<br><br>Transport to schools	Researcher
In-service the teachers about nutrition using DOH materials			
Distribute hard and soft copies of healthy eating from DOH			
Review and discuss exercise activities stipulated in the Life Orientation and Life Skills curriculum			
Guide the teachers on how to implement the designed structured exercise			
**Objective 8**: To strengthen the implementation of the NSNP programme at two identified primary schools through in-service training by the end of 2022			

**Activity**	**Time frame /frequency**	**Resources**	**Responsible person**
To communicate and distribute NSNP documents to all staff and school management	March 2022	Dietitian<br><br>NSNP Documents<br><br>Guidelines for Tuckshop Operators' documents	Researcher
To communicate and distribute Guidelines for Tuckshop Operators to all staff and school management			
